# Walking on eggshells: disabled people's management of emotions during everyday encounters in accessible parking spaces

**DOI:** 10.3389/fsoc.2025.1401620

**Published:** 2025-06-09

**Authors:** Vera Isabella Kubenz

**Affiliations:** Department of Social Policy, Sociology and Criminology, School of Social Policy and Society, University of Birmingham, Birmingham, United Kingdom

**Keywords:** disability, accessibility, affect, emotions, encounters, critical disability studies, parking

## Abstract

This paper explores how disabled people manage their own and other's emotions during encounters with strangers in accessible parking spaces in a UK context. Due to their mundanity, the affective impact of encounters is frequently not considered in the move towards removing barriers to public space for disabled people. Understanding the energy and emotion work that goes into managing these affects therefore offers a crucial new perspective on how we understand what “accessibility” means. Situating my analysis at the intersection between the sociology of emotions and critical disability studies, I present data from 20 disabled interview participants in England on their experiences of accessible parking encounters. This includes a discussion of the impression management and emotion work required to navigate encounters in parking spaces, and the exclusionary impact these encounters can have over time. In the findings I highlight how considering relational and psycho-emotional aspects of disablism are crucial when understanding everyday oppression and offer a way to rethink the negative emotions arising from encounters as a collective rather than an individual experience.

## 1 Introduction

This paper explores the extent to which disabled people are managing their own and others' emotions when trying to navigate encounters with strangers while using accessible parking spaces in a UK context. These encounters can have a significant effect on disabled people's emotional experiences of being in public: “Trying to understand the complicated feelings which arise out of our everyday encounters with the world is central to the lives of all disabled people” (Keith, 1996, p. 70). Building on findings from 20 interviews I conducted with disabled adults on their encounters with strangers in accessible parking spaces (also known as “Blue Badge” bays), I consider how public encounters do not just result from difference but can make (a) difference (Wilson, 2017) through replicating and reinforcing power inequalities between non-disabled and disabled people.

Employing an interdisciplinary approach, I weave together theories from the sociology of emotions with critical disability studies to demonstrate how thinking about affect may help us understand experiences of disability in a contemporary UK context. My approach is informed by an explicitly feminist and queer methodology which highlights how emotions play a crucial role in how people rationalise and make decisions when confronted with difficult situations (Ahmed, 2014; Hochschild, 2020). By conceiving of these emotions as a relational rather than a personal phenomenon, a focus on affect thus “offers a way of thinking about subjectivity that is not tied solely to the psyche” (Gorton, 2007, p. 345). In particular, my research is underpinned by the social-relational model of disability. This model draws explicit attention to interpersonal barriers by defining disability as

“a form of social oppression involving the social imposition of restrictions of activity on people with impairments and the socially engendered undermining of their psycho-emotional wellbeing” (Thomas, 1999, p. 60).

A key feature of disabled people's exclusion in the social-relational model is psycho-emotional disablism, which restricts what disabled people can be as well what they can do (Reeve, 2004, 2008, 2015). Negative attitudes from others can therefore be just as effective in excluding people as physical barriers, particularly because of “the 'existential insecurity' associated with the uncertainty of not knowing how the next stranger will react” (Reeve, 2008, p. 40). Disabled people who have experienced psycho-emotional disablism during encounters can thus be left permanently ill at ease in public spaces.

My exploration of encounters is thus situated within a broader focus on disability as a relational phenomenon, reflecting how public encounters with strangers tend to reflect power imbalances in society (Valentine, 2008). Simultaneously, this paper contributes to the sociology of emotions by drawing attention to how disability can be created through the strong emotions that can arise during and from interpersonal encounters. Specifically, I explore the relationship between affect and action, with disabled people feeling the need to act on the anxiety, uncertainty, and anger present in accessible parking spaces by managing themselves and others. Disabled people are thus always proverbially “walking on eggshells” in having to assess the risk of the current situation. I build on feminist affect theory which has highlighted both the productiveness of emotions and their power to not just replicate but heighten the “othering” of marginalised groups (Åhäll, 2018; Ahmed, 2014; Gorton, 2007). I link these theories to cultural theories of emotions in order to highlight how “culture conditions our emotional experiences and expression” (Bericat, 2015: 499) while at the same time replicating and reinforcing a culture in which disabled people are always regarded with suspicion. This includes drawing attention to the considerable amount of time and energy that goes into navigating the constant “anticipation of risk” (Burch, 2021, p. 151).

In this introductory section, I explore how Goffman's (1986) concept of stigma has been transferred into a twenty-first century context to explain how stigma is employed at an institutional level to replicate hierarchies of impairment. I then explore how both Goffman's impression management (Goffman, 1972, 1990) and Hochschild's (1979) and Hochschild (2020) concepts of emotion work and “feeling rules” can apply to how disabled people manage the emerging power balances in interactions with strangers. Finally, I also draw on Ahmed's (2014) conceptualisation of “sticky” affects to explore the intersections of emotions, encounters, and public space for disabled people. In the methods section, I give a brief overview of my use of critical disability studies and queer “scavenger” (Halberstam, 2011) methodologies, as well as my approach to data collection and analysis. My findings are structured into three sections, exploring the experience of being under constant surveillance by oneself and others; the need to expend emotional energy to manage potential or actual encounters; and the cumulative impact of relentless abjection and uncertainty. I then offer a discussion of how these findings can help us understand the psycho-emotional impacts of ableism as an integral and shared experience that is central to the disability experience in contexts of austerity and abjection, as found in the UK. I conclude with a challenge to how “accessibility” is conceptualised, as there can be no truly equal access if disabled people continue to face considerable “hassle” (Timm, 2002) and hostility in public spaces.

### 1.1 Stigma and hierarchies of impairment

Stigma is a key concept in understanding the continual marginalisation of disabled people in contemporary society. Stigma draws attention to the relationality of power, relying on both “the normal [sic!] and the stigmatised” to play their part in rendering the stigmatised person as inferior (Goffman, 1986, p. 33). According to Goffman's seminal work on stigma, this results in encounters being often awkward and uncertain, as the stigmatised can never know “how normals [sic!] will identify him [sic!] and receive him [sic!]” (Goffman, 1986, p. 18). Emotions are thus integral to the stigma process (Brown, 2013). While Goffman's work provides a useful starting point in thinking about how power relations play out in encounters and what may be the resulting affects, his work has been frequently criticised within disability studies as lacking criticality and naturalising rather than challenging stigma relations (Abrams, 2014; Coleman-Fountain and McLaughlin, 2013; Oliver, 1996).

Tyler's (2020) reconceptualisation of stigma offers a useful revitalisation in order to address how stigma operates simultaneously at personal and political levels in the context of twenty-first century Britain. Tyler's stigma recognises how stigma is always intricately connected to broader issues of social and economic power and hierarchies:

“while experienced intimately through stigmatising looks, comments, slights, remarks made in face-to-face or digitally mediated encounters, [stigma] is always enmeshed with wider capitalist structures of expropriation, domination, discipline and social control” (Tyler, 2020, p. 17).

In particular, stigma in this context is inextricably linked to government and media discourses to justify welfare reform, which have positioned the majority of disabled people as “fakers” and “scroungers”, pitted against a small minority of “legitimate” disabled people who are deserving of support (Briant et al., 2013; Garthwaite, 2011; Hughes, 2015; McEnhill and Byrne, 2014). The division of disabled people into “deserving” and “undeserving” is underpinned by disability hierarchies, which suggest that some impairments are more likely to be perceived as legitimate than others. In Deal's (2003) research on hierarchies held by non-disabled people, wheelchair use was seen as the most recognisable and acceptable way to be disabled. Similarly, Briant et al. (2013) found that people with physical impairment and/or sensory impairments were far more likely to be perceived as legitimate by both the media and the public. In contrast, people with impairments including mental health conditions, chronic pain, obesity, or substance dependence were seen as particularly likely to be “cheating the system”. Using a “divide and conquer” approach, stigma against those perceived as not deserving enough results in abjection of disabled people “as a mechanism of governance through aversion” (Tyler, 2013, p. 37) and has enabled successive governments to move ahead with cutting disability benefits with minimal public resistance. Understanding stigma as a deliberately created means of controlling populations through negative emotions is thus key to understanding the broader affective environment around disability in contemporary Britain.

### 1.2 Impression management and emotion work

Interactions with strangers often require significant work and active management in terms of how one is perceived and relates to the other person. The idea impression management is another important aspect of interpersonal encounters in public spaces first emerging from Goffman (1990). While everyone manages their self-representation when interacting with others, the power relations underlying encounters between disabled and non-disabled people mean that this impression management can be particularly fraught and burdensome for disabled people. Managing others' impressions often involves a performance of an “idealized” version of what the other person expects to see (Goffman, 1990). Recent work applying Goffman's work to disability has highlighted that disabled people may employ these management techniques strategically to negotiate difficult interactions (Scully, 2010; Wechuli, 2024). In the case of an encounter where the “legitimacy” of someone's impairment is being questioned by the other person, this can involve performing a “stereotypical” presentation of disability to make it more easily recognisable. What Siebers (2008) terms “masquerade”, i.e. exaggerating a limp or using a mobility aid more than strictly required, can be one way to manage the requirement to “look disabled” in order to be deserving in accessible parking spaces, particularly given the considerable suspicion around “fakers” prevalent in British society. The performance of disability is thus a survival mechanism (Scully, 2010; Wechuli, 2024). However, appearing to “look disabled” alone is often not enough to satisfy suspicions, as disability stereotypes also prescribe how a disabled person should act. Incompetence and inferiority are thus integral aspects of the disabled role:

“the cripple [sic!] must be careful not to act differently from what people expect him to do. Above all they expect the cripple to be crippled; to be disabled and helpless: to be inferior to themselves, and they will become suspicious and insecure if the cripple falls short of these expectations. It is rather strange, but the cripple has to play the part of the cripple.” (Goffman, 1986, p. 88)

The reproduction of power imbalances is thus crucial to encounters. Building on Goffman's work, Hochschild's concept of “feeling rules” describes how interactions with others are guided by “what is emotionally due another person” (Hochschild, 2020, p. 19). Feeling rules are infused with unequal power relations. While Hochschild's original work focuses primarily on gendered power dynamics in an employment context, it has since been adapted to explore how disabled people are often expected to take responsibility for how we make others feel (French, 1994; Garland-Thomson, 2006; Keller and Galgay, 2010; Pritchard, 2021; Scully, 2010). This often means performing significant emotion work (Hochschild, 2020), modifying one's own feelings and behaviour to remain polite and deferential even when the other person is not, for example not getting angry when being stared at, patronised, or asked intrusive or personal questions. Key to being disabled in public is not just having an easily recognisable impairment, but to put in the emotion work needed to perform the role of the “good” disabled person who is always grateful, good-humoured, and compliant (Cahill and Eggleston, 1994; Keith, 1996; Reeve, 2006, 2008; Wilkin, 2020).

While the performance of emotion work can be extremely draining, refusing to abide by the established “feeling rules” by resisting stereotypical expectations can be equally fraught. Challenging others on their harmful assumptions can potentially result in extreme reactions from the other person, including outright hostility and aggression from the stranger (Burch, 2021; Morris, 1991; Siebers, 2008). Disabled people who do challenge others may feel guilty about provoking them into anger, or worry that this challenge may have negative consequences for other disabled people in future encounters (Cahill and Eggleston, 1994; Morris, 1991; Tregaskis, 2003). The emotions arising from encounters thus have the power to influence how disabled people navigate public space.

### 1.3 Affect, encounters, and space

The affects resulting from impression management and emotion work in interpersonal encounters are not just the final outcome of an unpleasant interaction, but are productive, shaping the encounter as it unfolds. The idea of emotions as affective practises that are “always ‘turned on' and ‘simmering', moving along” (Wetherell, 2012, p. 12) is key to understanding how encounters cannot just produce negative emotions such as anxiety, but also spur disabled people on into taking action. Thinking about emotions not as individually held feelings, but as affects which “stick” to both individuals and spaces (Ahmed, 2001), can help illuminate why accessible parking spaces are particular hotspots for intense emotional encounters. As one of the few spaces where disability is expected in public life, they draw attention to disability and thus serve as a location where societal prejudices of disabled people as either helpless, “vulnerable” recipients of charity, or as feckless scroungers, are concentrated. While accessible parking has been exempted from public sector cuts, the emotions of resentment and envy associated with government “scrounger” rhetoric (Hughes, 2015) nevertheless stick to disabled bodies. Conversely, emotions felt by disabled people such as anxiety and fear can become “sticky”, particularly in spaces where hate was previously experienced, creating a negative “affective atmosphere” (Burch, 2021, p. 65) which means they can never be at ease in these spaces.

Spatiality is thus crucial in exploring encounters, with emotions, space, and the people within it mutually constituting. Conversely, encounters play a key role in shaping disabled people's experience of space (Cahill and Eggleston, 1995; Morris, 1991). A defining feature of encounters is that they are naturally uncertain and ambiguous (Wilson, 2017), with the possibility of escalation at any point. It is precisely because encounters are common and everyday occurrences that they are impactful. Hate and abjection of disabled people in public space is not extreme or exceptional, but a commonplace phenomenon in disabled people's everyday lives (Burch, 2021; Hall, 2019; Hall and Bates, 2019; Piggott, 2011; Wilkin, 2020; Hollomotz, 2013). Recent research on disability hate crime highlights the importance of space to acts of harassment and violence, with public transport and accessible parking bays emerging as particular hotspots (Hall, 2019, 2024). Occupying public space is thus not a neutral act, but rather, spaces are fundamentally social, both shaping and being shaped by the people within them (Lefebvre, 1991). It thus requires a great deal of care and attention to navigate certain spaces.

Another way in which space and affects are mutually affecting is through the emotional impacts of systematic exclusion. Encounters are effective in stirring up negative emotions about disability precisely because disabled bodies are still often absent from public spaces. Perpetual inaccessibility in the public built environment continues to exclude disabled people on a physical level (Hall and Bates, 2019; Hall and Wilton, 2017; Imrie, 2001). Disabled people thus become Ahmed (2000, p. 56) “stranger”, a body that is recognised as out of place and fundamentally other to themselves. While accessible spaces such as parking spaces are seemingly a solution to the issue of structural inaccessibility, it has been argued that segregating accessibility into dedicated spaces in fact perpetuates “othering” by normalising inaccessibility elsewhere (Reeve, 2014, 2008; Slater and Jones, 2021; Titchkosky, 2011). The presence of signage such as the International Symbol of Access (better known as the wheelchair symbol), which marks accessible spaces, further shapes the encounters and affects present, but marking out which bodies are and are not welcome in this space (Slater and Jones, 2021). In a context where disabled people are under constant suspicion of “faking”, this signage can therefore leave disabled people who are not visible as wheelchair users anxious about potential challenge from others. Accessibility is thus not a fixed state but shaped in large part by the interactions with others and their associated affects.

## 2 Methodology and theoretical approach

### 2.1 Framework

Employing a critical disability studies (CDS) lens, my research takes an “eclectic approach” (Meekosha and Shuttleworth, 2009) to interdisciplinarity, bringing together the sociology of emotions, psychology, human geography, and cultural studies to understand encounters. CDS thus opens up the possibility for multiple epistemological approaches and understandings of disability to co-exist and sometimes even merge (Meekosha and Shuttleworth, 2009; Flynn, 2017; Egner, 2017). My framework for this research is informed particularly by feminist and queer emancipatory methodologies.^1^ My interest in the impact of public encounters, especially in accessible parking spaces, stemmed from my own experience of using these spaces as a disabled person. I experienced these spaces as anything but “accessible”, and rather as places where I felt I needed to modify my own behaviour in order to manage or avoid actual or potential encounters. Using the feminist lens of the personal as political (Morris, 1992) I sought to make sense of my own emotions through research. My research is thus deeply indebted to the feminist disabled theorists who pioneered writing about psycho-emotional disablism and the impact of interpersonal encounters (Keith, 1996; Morris, 1991; Reeve, 2008; Thomas, 1999).

In order to explore encounters in all their complexity, I employ a mixed-method approach which for the answering of multidimensional research questions (Collins, 2015). Mixed methods approaches are also frequently employed in feminist and intersectional research approaches, allowing room for contradictions and multiple ways of knowing (Cram and Mertens, 2015; Hesse-Biber and Griffin, 2015; Hankivsky and Grace, 2015). Likewise, critical and transformative designs often include a mixed method approach that aims to centre marginalised voices (Creswell and Plano Clark, 2018; Cram and Mertens, 2015; Plano Clark and Ivankova, 2016). This can include the use of quantitative methods, which can be compatible with empowering approaches (Cornelius and Harrington, 2014) and have been employed effectively in feminist research to “dismantle the master's house” (Hesse-Biber and Griffin, 2015, p. 76). Transformative mixed methods research thus tend to have a “de-disciplining” effect (Hesse-Biber, 2015, p. xxxiv), with a tendency to focus on transformative concerns over epistemological or disciplinary conventions. In this way, my approach can be likened to a queer “scavenger” methodology, which puts the centring of marginalised voices above epistemological congruity:

“uses different methods to collect and produce information on subjects who have been deliberately or accidentally excluded from traditional studies of human behavior. The queer methodology attempts to combine methods that are often cast as being at odds with each other, and it refuses the academic compulsion toward disciplinary coherence.” (Halberstam, 2018, p. 13)

Intersectionality is a central focus for me within in this research, in line with concerns within critical disability studies to understand how disablism intermeshes with other forms of prejudice including racism, sexism, and homo-/transphobia (Schalk and Kim, 2020; Siebers, 2008). This has informed my sampling strategy in aiming to recruit participants with diverse experiences and identities. I have also sought to centre during my analysis how participants reflect on the impact of their intersecting identities. Another feature of my approach which is immersed in both queer and postmodern approaches which is my desire to resist and where possible, actively deconstruct, binaries (Egner, 2017; Halberstam, 2011) and to disrupt the status quo by going against conventions (Kafer, 2013; Slater, 2013). Some binaries challenged in this paper include the ideas of accessible/inaccessible, deserving/undeserving, and “looking”/”not looking” disabled. Challenging these binaries is central to highlighting the murkiness, ambiguity, and uncertainty disabled people often feel when they do not fit into these neat categories.

### 2.2 Data collection and analysis

The findings presented in this paper come from data collected in 20 semi-structured interviews, which formed the second phase of the mixed-method project. Mixed-methods approaches are common in feminist designs (Creswell and Plano Clark, 2018; Hesse-Biber and Griffin, 2015), and I employed this approach based on my commitment to capturing the nuances and complexities of encounters. Throughout the research process, I worked with an advisory group of 7 Blue Badge holders, to ensure that the research reflected the experiences and concerns of other disabled people as well as myself. The advisory group members were consulted before the launch of each data collection phase as well as afterwards to sense-cheque the results. They were compensated for their time and expertise with an honorarium. Ethics approval for each phase was gained from the relevant institutional review board. Given my own experience with such encounters, I was particularly aware that they may be distressing, so participants were provided with a list of resources for practical and pastoral support during each stage.

The 20 interviewees were recruited from a pool of over 300 disabled people who had previously completed a survey on Blue Badge encounters during the first phase of the research. This survey was shared through social media (Twitter, LinkedIn) and sent to 178 Disabled People's Organisations in England. It was open to disabled people aged 18+ resident in England who currently or in the past held a Blue Badge for themselves. Participants for the follow-up interviews were selected from those who had indicated their interest in this during the survey. Invitees were chosen using a purposive, heterogenous sampling approach (Aidley and Fearon, 2021) to ensure I collected as many diverse experiences as possible. Interviewees were invited in stages to cover a variety of impairments, ages, genders, ethnicities, sexual orientations, and types of encounters experienced. In total, I interviewed 10 men, 9 women, and 1 non-binary person. 16 participants were white, 1 was Asian, 1 had a mixed ethnic background, and 2 did not give their ethnic background. Interviews took place online via videocall, by telephone, or by email, depending on each participant's preference. Participants were also asked to self-define whether their impairment was visible. Most participants had an always visible impairment (11 out of 20), 6 had a sometimes visible impairment, and 3 had a never visible impairment. Interviewees were invited to review the transcripts after the interview and to choose their own pseudonyms. Table 1 provides a full summary of the interview participants' characteristics.

**Table 1:** Overview of interview participants.

**Participant pseudonym**	**Gender**	**English region**	**Age range**	**Ethnic background**	**Sexual orientation**	**Impairment type(s)**	**Impairment visibility**
Amir	Male	Midlands	18–29	Asian	Bisexual	Mobility, mental health	Sometimes
Anna	Female	South	40–49	White	Straight	Chronic illness, mental health, mobility	Sometimes
Charlie	Non-binary	South	30–39	White	Lesbian	Chronic illness, mobility	Sometimes
Chris	Male	Midlands	60–69	White	Straight	Blind, chronic illness, deaf, mobility	Always
Elizabeth	Female	South	70+	White	Straight	Mobility	Always
Emma	Female	Midlands	50–59	White	Bisexual	Chronic illness, mobility	Sometimes
Frank	Male	Midlands	70+	White	Straight	Deaf, mobility	Always
Frederick	Male	South	70+	White	Straight	Chronic illness, deaf, mental health, other	Always
George	Male	Midlands	50–59	White	Straight	Chronic illness	Always
Henry	Male	London	40–49	White	Gay	Chronic illness, mobility	Never
Isabella	Female	London	30–39	White	Bisexual	Chronic illness, mental health, mobility	Always
Ivy	Female	North	18–29	White	Bisexual	Chronic illness, mental health, neurodivergence	Sometimes
John	Male	Midlands	60–69	White	Straight	Mobility	Always
James	Male	London	50–59	White	Gay	Chronic illness, mobility	Always
Julie	Female	North	50–59	White	Straight	Chronic illness, mobility	Always
Katie	Female	South	18–29	White	Straight	Chronic illness, mobility	Sometimes
Louise	Female	South	50–59	Unknown	Straight	Chronic illness	Never
Lydia	Female	North	40–49	Mixed background	Straight	Chronic illness, mental health, neurodivergence	Never
Richard	Male	North	70+	Unknown	Straight	Chronic illness, mobility	Always
Will	Male	London	30–39	White	Straight	Mobility	Always

The approved transcripts were analysed using Braun and Clarke's (2022) approach to reflexive thematic analysis. This widely used analytical approach is about critical and questioning engagement with qualitative data, seeking to capture “nuance, complexity and even contradiction” (Braun and Clarke, 2022, p. 7). Further, its centring of reflexivity in the analysis is embedded within feminist research approaches which value the subjective experience and skills of the researcher (Braun and Clarke, 2021). This allowed me to bring in my own experiences of accessible parking encounters, and reflect on how they shaped my own analytical choices and interests (Braun and Clarke, 2022; Trainor and Bundon, 2021). In particularly, I realised that I was particularly interested in interrogating the spoken and unspoken contradictions within my participants' account. My analysis process for the reflexive TA closely followed Braun and Clarke's (2022, 2006) six-step process of (1) Dataset familiarisation, (2) Data coding, (3) Initial theme generation, (4) Theme development and review, (5) Theme, refining, defining, and naming, and (6) Writing up. Through this process, I generated four themes with a total of ten subthemes. The findings discussed in this paper come from four subthemes relevant to the field of emotions and impression management, titled “Hierarchies and legitimacy”, “Walking on eggshells”, “Abjection and hate”, and “Slow death and exhaustion”.

## 3 Findings

### 3.1 (Self-)Surveillance and impression management: “that balance is always there”

The first way in which disabled people manage emotions in accessible parking spaces relates to the way in which we manage our own behaviours and appearances to defuse or avoid encounters. This is often shaped by what Manji (2017) terms “sousveillance”, a bottom-up approach to surveillance that encourages communities to police each other through acts of vigilante enforcement. Media reporting on taxpayer's money being squandered by benefits scroungers and cheats creates a sense of entitlement amongst the non-disabled public to cheque whether disabled people are really “legitimate” and deserving, as illustrated by Amir's experience:

I usually sit in a seat in the car. And my wheelchair gets folded up in the boot. I don't sit in the wheelchair in the car. So, if you walk past the car window what you see is a, quote, “normal looking person”. And people will…. will say things to me or my parents. Along the lines of “Why are you parked here?” And if… it might be a bit less polite. The things they usually say are, “Why the fuck are you parked here?” That's the kind of things people will usually say. “You don't need that space.” “It's for real disabled people”. “You don't look disabled.” Because while I'm sitting in a car seat… I mean, I look… “ordinary”. I hate this term, but it's kind of, a good description, I think. (Amir, Asian man with always visible impairment, age group 18-29).

A particularly frequent question my participants received from strangers is “What's wrong with you?” The question is “othering” through reinforcing the medical model assumption that disability as a defect or a “problem” that makes someone different from a “healthy”, “normal” person. It is also bound up in power relations. The surveillance of disabled bodies becomes a form of disciplinary power (Foucault, 1991), enacted by governments and replicated by the public upon disabled people to ensure only the “right” kind of disabled person is able to access certain accommodations, welfare payments, or accessible parking. Disabled people are thus under pressure to ensure they are always perceived as “legitimate” by strangers in order to access spaces.

Being perceived as “not looking disabled” can be a considerable source of anxiety. In an environment of suspicion and distrust of disabled people, those who feel they do not fit the expected image can feel constantly on edge about a potential confrontation. Hierarchies of disability lead to a narrow view of how disability should present, and rejection of anyone who does not adhere to this stereotypical image. The stereotype of a typical disabled person has previously been conceptualised as either a “young, male, white wheelchair user” (Shakespeare, 1996, p. 195) or an older wheelchair user (Reeve, 2008). My participants were acutely aware of this stereotype and the potential consequences of not “looking disabled”. Younger disabled people particularly felt they were frequently targeted because of their age, and several female participants spoke about never travelling alone due to feeling unsafe. Even several of my wheelchair-using participants, such as Amir, were subject to intrusive questions or looks, usually before they had got their wheelchair out of the car. All disabled people are thus potentially at risk of being questioned in accessible parking spaces, and were often acutely aware the different ways in which they were potentially inconsistent with a stereotype, as shown in Emma's interview:

I think they seem to think that Blue Badge holders are wheelchair users, which is not the case, and I don't know… if I get targeted because I'm a Goth. You know, I dress like a Goth. I am a Goth, and have red and black hair, and I don't know if… because I look quite different that I'm targeted and… I don't know, from talking to all Blue Badge users, we're all targeted. We're all told, “I don't think you should be in that space”, when it's got nothing to do with them. You know I do feel there's a real policing by the public of the Blue Badge spaces, Blue Badge holders. Um…yeah. But I just think, I just think you can't look like that. You can't look like me, you know, from a subculture. You can't be young. You can't not be in- not use a wheelchair. You can't not have a visible disability. (Emma, white woman with sometimes visible impairment, age group 50-59).

Emma's storey illustrates the many different ways in which she understands herself as not matching what a stereotypical disabled person should look and behave like. Incongruence is policed heavily precisely because of its potential to destabilise the disabled/non-disabled binary which underpins ableism (McRuer, 2002). Disability is required to be “fixed, permanent, internally homogenous and, moreover, oppositional” to the non-disabled body (Shildrick and Price, 1996, p. 95). Experiencing these confrontations in addition to the inescapability of “deservingness” discourse in wider society alongside means that we may internalise these discourses. Some of my participants who were closer to the top of the legitimacy hierarchy (e.g., older white men with physical impairments) spoke about sometimes doubting whether others were legitimate. Charlie on the other hand, had only recently transitioned to using a wheelchair and used accessible parking primarily for the extra width. They felt that their use of accessible parking bays was not just shaped by encounters with others, but also by self-doubt about whether they were “deserving” enough to use the bays:

So you go into a spiral […] with some of that kind of challenge over looking young. And relatively healthy until they saw something. Or… you got the glares, you got the… the “Shouldn't you leave that bay? Shouldn't you leave that parking for somebody who needs it?” with the, you know, the implication being that you don't need it. And it's still some of that fuel of my knowing I don't need to be so close to the storefront, I can feel quite self-conscious about using blue badge parking. Especially when it's very clearly blue badge parking that's mostly full because what if somebody who does need to be near the store needs it? But that isn't a confrontation I'm having. That's still that relic of the “Perhaps you're not disabled enough…” voice in the back of your head. (Charlie, white non-binary person with sometimes visible impairment, age group 30-39).

The anxiety and doubt experienced by disabled people when worrying about being confronted meant that many of them took action in order to reduce the risk of confrontation. Like Foucault's panopticon, those under constant surveillance internalised this practise and managed their own behaviour to adapt to the required standard (Burr, 2015; Foucault, 1991). This included the employment of impression management skills to try to convey recognisable “disability” to others. Two of my participants, who were both young women under 30, spoke about using masquerade to do this:

But there's definitely things I do to protect myself like I said, I use my walking stick when I'm on my own to get from the front of the car to the back, which I wouldn't do when someone's with me. And… I think… [pauses] sometimes my limp is probably a bit more pronounced when I am on my own as well than when I'm with somebody. And I think it's things like that, that it's just… trying to stop other people from kind of… judging me. And yeah. (Katie, white woman with sometimes visible impairment, age group 18-29).

For both Katie and the other participant, masquerade was a tool to reduce the potential risk of an encounter and helped to manage the anxiety they felt as a result. However, not all self-management necessarily involved the performance of an “idealized” version of disability. A few of my other participants felt that being *too* visible as a disabled person produced a different kind of risk, that of being targeted for disability. Julie (a white woman with always visible impairment, age group 50–59), who had experienced a hate crime perpetrated by teenagers who assaulted her while in an accessible parking space, felt that the wheelchair stickers on her car where part of the reason why she had been targeted. Similarly, Emma felt hesitant about using her walking aid in public because it would mark her out “as vulnerable” and potentially an easy target for harassment:

But I've noticed that having that walking stick changes you from an invisible disability to a visible disability. But the other thing that concerns me about this is, it also makes me look a bit more vulnerable. So I'm always a little bit wary. But now I use my stick whenever I go out, because one of my knees gives way. So I'm trying to attend upon the deck again. And I just kind of… I'm just really careful about getting that balance between… I need to look like I've got a disability, because, you know what, I might need to sit there, or I might need to park there or do whatever. But also I don't want to feel quite so vulnerable. And yeah, that always… that balance is always there. (Emma, white woman with sometimes visible impairment, 50-59).

These storeys highlight how managing visibility of one's impairment is an ongoing and complex process for many disabled people. It requires much more nuance than captured in Goffman's type of impression management performed by us all, with careful judgement and constant re-evaluation of the situation in order to gauge the “risk” of a confrontation. This leads to parking spaces being associated with being spaces of anxiety for many disabled people, as well as taking considerable energy due to the high demand of continually reflecting and assessing on one's own and other behaviours. However, the emotional and physical costs of self-surveillance are just one part of the storey and are added to the need to manage interactions with others, which will be explored further in the next section.

### 3.2 Emotion work and feeling rules: “you've got to be the bigger person”

As well as managing oneself during an encounter, my participants also performed emotion work to manage the interaction with the other person involved. Overall, many of my participants were strongly guided by a sense of needing to remain polite and non-confrontational in Blue Badge bays. This was the case even where the disabled person initiated an encounter; for instance, when challenging someone who was using a parking bay without a permit. Elizabeth, who out of principle challenged people who abused accessible parking bays without a Blue Badge, discussed how she used politeness as a tool to manage the risk of an encounter escalating:

I will put notes on people's car and just say “Whoops, you've forgotten your Blue Badge.” Or I say to people, you know, if the person's there, I'll say “Ooh. Have you forgotten your Blue Badge?” I will try that angle. Because yes, people do get very, very stroppy and very aggressive. And I don't want to sort of rile them up. So I think if you sort of approach it from that angle, you're giving them an opt out. Or you're maybe embarrassing them. < Interviewer: Do you find that most successful than direct confrontation?> Elizabeth: Um… I don't find that either works, to be honest. I've tried both. And yeah, people, if people are gonna abuse a Blue Badge bay, they will. (Elizabeth, white woman with always visible impairment, 70+).

Storeys such as Elizabeth's highlight the extent to which emotion work is bound up with power relations (Hochschild, 1979). My participants were acutely aware of the expectations of disabled people to be polite and well-behaved in public and suppress the urge to show one's frustration or anger. As my participant Anna (a white woman aged 40-49 with sometimes visible impairment) put it: “You do feel like being rude back sometimes. You've got to be the bigger person really, you know, not let them get to you.”

The affective atmosphere of anxiety permeating accessible parking spaces was a key factor in shaping this very careful approach of “walking on eggshells”, with my participants perceiving this as a particularly perilous and uncertain space where a confrontation could escalate at any moment. While the term “vulnerable” has rightly been criticised for being assigned to disabled people as a way of reinforcing medical model stereotypes of disability (Finkelstein, 1998; Garland-Thomson, 1997; Hughes, 2007; Ralph et al., 2016), some of my participants used this term to describe how they felt in this situation and why they chose to avoid confrontation rather than challenge the other person about their poor behaviour:

I just tend not to look at people if I think that somebody's… you know. And I do see sometimes that there are a couple of people arguing and I think, well, I don't really want to get involved because I feel vulnerable. And being in a wheelchair, if somebody tipped me out of my wheelchair and took my wheelchair away, I wouldn't be able to move. You know, because I can't physically stand and I can't crawl or move like that… So I tend to avoid stuff because I'm inwardly nervous. I'm quite a strong character, but then I don't like getting into confrontation with people because I don't want to deal with the aftermath, if that makes sense. So I try and avoid it as much as I can. (Chris, white man with always visible impairment, age group 60-69).

The effort that goes into managing encounters, then, is not just the emotion work of suppressing one's true feelings, e.g., of annoyance or anger at the person misusing the parking space, and reflecting the expected emotions prescribed by feeling rules. In addition, considerable work goes into “reading” the situation and the other person to weigh up what is the best strategy for handling a particular encounter. This complex process involves a split-second assessment of the situation, including determining one's own energy levels, gauging how the other person may react (e.g., will they be receptive or potentially aggressive), and then choosing how to manage the encounter. Charlie, who was naturally assertive, described the assessments they make before choosing whether to challenge someone about their attitudes:

I am a little confrontational… There are people who I will avoid. Getting into that one with…. It tends to be about the body language. It's not specifically about gender, race, or sex. It's “How much of a fight are they looking for?” If they're being snide but it's snide in the “I'd like to get into an argument with you to prove a point or something”, that one I will just try and ignore it. Um… If the person having a go at somebody else in the blue badge is going to be aggressive, it will be a case of me looking for like, is the shop security or something nearby? Um… rather than necessarily getting into it myself. But I think I am probably a little bit more arsey [sic!] than some people would because of the how and the why of - like previous experiences and stuff. (Charlie, white non-binary person with sometimes visible impairment, age group 30-39).

Charlie's approach to weighing up the risk of confronting another person lays bare that choosing how to react in an encounter is often based purely on instinct. As Scully (2010) asserts then, there is no right or wrong way to handle an encounter, as disabled people do not have a genuinely free choice in how to react. While disabled people can choose “emotional deviation” (Bericat, 2015, p. 499) to break “feeling rules”, asserting oneself comes at potential risk of one's own safety and disabled people who do challenge may feel also guilty about provoking anger in others, or worry about their behaviour having negative consequences for other disabled people in the future (Cahill and Eggleston, 1994; Morris, 1991; Tregaskis, 2003). The power asymmetry that underpins “feeling rules” means that disabled people cannot win, even when the other party does not adhere to the same feeling rules, for example through making patronising comments, invading the disabled person's personal space, or asking intrusive or personal questions. For example, when Ivy lost her temper with a woman who questioned the legitimacy of her Blue Badge, the confronter became offended and defensive, rather than reflecting on the inappropriateness of her own behaviour:

And I said, you know, like “It's none of your business.” I swore a bit. I was like, “Leave me alone. This is nothing to do with you.” And then she reacted really badly, like “Ohhh… well, I have to check!” No, you don't have to check! [laughs] Like, you're not a warden of the car park! It's not your… And I said something where I was like “Well, who are the fuck are you, the Blue Badge police?” And she got really offended. And I was like, “Look, I'm going to be angry because you just literally confronted me when I'm just trying to get to my appointment.” Sorry, am I allowed to swear, is that okay? (Ivy, white woman with sometimes visible impairment, aged 18-29).

During the interview with Ivy, she clearly felt very guilty about the incident, telling me she regretted her reaction and wish she had handled it differently: “I do regret shouting at that woman and swearing because that was rude. My mum raised me better, you know.” When I asked Ivy if she thought the woman would have listened to her if she had explained herself more calmly, Ivy conceded the conversation would have probably played out in much the same way. This highlights how breaking “feeling rules” can be difficult in its own right, with going against the norms of politeness and public order (Goffman, 1972), and in Ivy's case, against what our parents have taught us, resulting in feelings of guilt.

Ivy was not the only participant who struggled to control her reaction to someone else's inappropriate behaviour. The abjection disabled people experience in accessible parking spaces can feel intensely personal. As a result, several of my participant found it difficult to manage the anger they felt and not lose their temper. Anger is one of the emotions antithetical to disabled people's expected presentation as always cheerful and grateful: “We are certainly not supposed to get angry” (Keith, 1996, p. 81). Managing anger, then, was a central aspect of the emotion work that takes place in parking spaces to many of my participants. For example, Will discussed how he was happy with his “performance” of containing his anger during an encounter that had the potential to turn into a violent situation:

Yeah, particularly that one with the guy who nearly got into a fight, which I thought was a bit odd. Uh… I was quite actually pleased with that one that I reacted how I did cause I… I didn't react. Sometimes I can react a bit agg- a bit angrily to people, but that one I managed to stay really calm because he got very, very angry and was literally coming up right in our face and saying “Do you want to fight about it?” And we were like - I was like, “Well, I don't wanna fight about a car parking space.” And yeah, I was quite happy with my response to that one. Sometimes, yeah, if I argue, it can just stay on my mind and kind of run over and over, and what might have happened, kind of thing? (Will, white male with always visible impairment, age group 50-59).

The requirement to manage both one's own emotion and those of others mean disabled people need to perform considerable work to be able to exist in public spaces (Burch, 2021; Scully, 2010; Thomas and Sakellariou, 2018; Watermeyer and Swartz, 2008), in addition to and going far beyond the kind of impression management performed by all of us on a daily basis. This is not only physically and emotionally exhausting but comes associated with the constant worry of making a wrong decision which could lead to an escalation of the situation. The resulting existential insecurity (Reeve, 2008) is reinforced through the cumulative impact of encounters over time, and this will be explored in the final section of this analysis.

### 3.3 Microaggressions and slow death: “it's often not worth the hassle”

Negative encounters with strangers can encompass a wide variety of interactions. Recent research on disability hate has shifted to focusing on the full spectrum of these experiences, recognising that most incidents are not extreme acts of hate, but that low-level discrimination and abjection are pervasive everyday experiences for many disabled people (Burch, 2021; Hall, 2019; Hall and Bates, 2019; Piggott, 2011; Wilkin, 2020). Collectively, my participants had experienced the full range of the “continuum of hate” (Hollomotz, 2013), ranging from hate crime and physical violence to threats, verbal abuse, and to more subtle, passive-aggressive provocations, such as tutting and almost invisible stares.

While at least two of my participants recounted clear hate crime incidents in which the police had been involved, many others had experiences that in themselves could have seemed innocuous, but for the participants were deeply upsetting. Everyday encounters often took the form of microaggressions, low-level and subtle behaviours which intentionally or unintentionally “communicate hostile, derogatory, or negative […] slights and insults to the target person or group” (Sue, 2010, p. 5). Anna described how being stared at while getting out of the car in a Blue Badge bay was an encounter that stayed with her, precisely because of the “respectability” of the man doing the staring, and her own perception of herself as visibly and therefore “legitimately” disabled due to her use of mobility aids:

Yeah, I still think about the person that stared at me the most. And I felt it especially as it was in quite an affluent area of our city. And I thought… I kind of presumed, and this is me showing presumption, he looked well-dressed, he looked respectful. And he just stood there and stared at me completely Ignorantly, almost as if… and you could see I was on crutches. Even when the car pulled in, the crutches were in the front seat with me. So, because I need to get out quite quickly, we couldn't wait to get them from the back of the car. You could actually see above the door line that I was holding the crutches up, off my knees. (Anna, white woman aged 40-49 with sometimes visible impairment).

Anna's storey highlights how the feeling of anxiety stemmed not just from being stared at, but also from the dissonance between Anna's perception of the visibility of her disability, and the challenge she nevertheless experienced. It is precisely the subtlety of microaggressions such as staring that contribute to disabled people experience of them as psycho-emotional disablism. Due to their uncertainty and ambiguity the disabled person may be second-guessing their own perception of the event, wondering if it really happened, or if they are overreacting. The insidious nature of microaggressions also makes it difficult for others to understand their true impact. James spoke about how his non-disabled friends and family did not fully appreciate the gravity of encounters and how much they affected him:

It's like everybody tunes out to it. “Oh, it's just - it's happened again.” Well, life's… and the actual kind of feelings that it can… trigger off. People don't want to hear about, you know, all that side of things. And I don't think they… I don't know that they get that. “Oh, it's just a parking space, Jim, and don't get so obsessed by it” or that kind of… and you think it's - but that's the thing, it's not just a parking space. It's whether or not I can do what you've just taken for granted. (James, white man with always visible impairment, age group 50-59).

Another key feature of microaggressions is their effect over time, leading to “death by a thousand paper cuts” (Nittle, 2019, p. 9). Many of my participants, including Anna and James, still vividly remembered and replayed particularly impactful encounters in their heads. Louise spoke about how she felt that an encounter that tainted a rare day out with her extended family had affected her in such a profound way that it intermeshed with her existing PTSD:

And, you know, it definitely put a dampener on the day and I kind of feel like if I could have erased that day, not had it, and, you know, done what we all do another time without that encounter, then that's great. You know. And… I mean… you know… I had a lot of things to you know, memories, and I try to not focus on these things, so I don't want that to be a lasting memory of the day, but it hasn't gone out from my mind and I think it's because…. it's actually created trauma. It was traumatic. And… so it's unfortunately stuck in my mind because you know, because he intimidated me, he was aggressive and so… so yeah, it's still here because one of my diagnoses is PTSD, so you know, it if a man… or someone is confrontational and aggressive and I feel the need to protect myself and… it creates a PTSD sort of cycle. (Louise, white woman with never visible impairment, 50-59).

While not all encounters are necessarily traumatic, Morrigan's (2017) conceptualisation of living with trauma as being like time travel is useful to understand the multiple temporalities involved in repeated encounters, as the trauma of past experiences shapes the possibilities for action in the present and the future. The lingering negative emotions associated from past encounters thus may contribute to the expectation of having further encounters (Mclaughlin and Coleman-Fountain, 2018).

The anxiety underpinning the need for impression management and self-surveillance discussed in the first findings section is always present in accessible parking bays, regardless or not whether an actual encounter takes place. Even when no encounter occurs, the possibility of one is always looming. Memories of encounters are thus a constant “absent presence” (Burch, 2021, p. 165), which disabled people have to actively address. Indeed, one of my participants had never experienced an overtly negative encounter, but nevertheless felt worried based both on her experience of negative encounters in other public spaces and from hearing about negative parking incidents from other disabled people:

Interviewer: You said you've mostly had positive interactions […] in what situation would you want to avoid an interaction? Is it just that you don't feel like talking to people or just..? Isabella: I suppose because it *could* be a negative interaction. And I, sort of still feel…. Um… from some people you know that in society, there is hostility and discrimination. And I suppose… um… perhaps I'm worried that something might happen, even though nothing has happened to me. I know that people can have some worrying and distressing interactions. And I wouldn't want to put myself in that position. (Isabella, white woman with always visible impairment, age group 30-39).

Isabella's cautious approach informed by her anxiety over a potential encounter at any moment illustrates how disabled people live “in a constant state of “questioning”' (Sue, 2010, p. 73), with accessible parking spaces just one of many locations where we can never feel fully secure. It also highlights how the anxiety and uncertainty associated with experiencing psycho-emotional disablism is not just an individual experience but takes on a communal nature with parking spaces acquiring notoriety among the disabled community as a space where we are particularly at risk. This highlights how encounters are not necessarily an individual, private event, but the affects resulting from microaggressions can be transferred between disabled people to create an atmosphere of fear and anxiety, always “linked to a wider sociopolitical context of oppression and injustice” (Sue, 2010, p. 96). Several of my participants shared storeys about encounters with other disabled people, either in person or through online forums and social media networks. This sharing of experiences was a crucial support mechanism to reduce the isolation and self-doubt inherent in psycho-emotional disablism for these participants, providing confirmation that it was not just all in their heads. However, as Isabella's comments shows, it could also result in “second-hand” anxiety from other's encounters. Encounters thus became a communally shared experience among disabled people, influenced by the knowledge that these kinds of events are commonplace in accessible parking spaces, and highlighting another way in which affects are constituted relationally between people and spaces (Ahmed, 2014; Lipman, 2006; Wetherell, 2012, 2015).

The knowledge that sooner or later an encounter is inevitable sentences disabled people to a form of slow death (Berlant, 2011) through ordinary and taken-for-granted everyday moments contributing to their wearing down as a group. Along with repeated encounters comes the realisation that our existence in public spaces is always at best conditionally tolerated and at worst there is a constant risk to our safety. The contingent acceptance of our presence by others in public can be just as effective as excluding disabled people from public spaces as physical barriers (Reeve, 2008). Many of my participants spoke about no longer going out due to past negative encounters, either temporarily after an encounter had occurred, although some limited themselves to essential journeys more permanently:

So I think this is part of the fact that sometimes it's easier not to go out than it is to go out. It's often not worth the hassle. I'd love to go to town and buy a hat for example. I want to buy it. I lost my hat, so I want to replace it. But It's so much trouble trying to get to the… to park outside the shop that… what is it, since October last year I've been planning to go but I won't go because it's too much hassle. So, yeah, I would say it's more of a “I avoid getting into that” situation. (George, white male with always visible impairment, 50-59).

While negative attitudes towards disability persist and their affects permeate public spaces, no space can be truly “accessible”. Rather, it puts disabled people in a no-win situation where we either limit our own access to public space, or need to perform significant management and emotion work to negotiate public spaces as a trade-off for the participation in public life that others take for granted.

## 4 Discussion

The experiences of my participants in navigating the affective landscape of accessible parking spaces highlights the difficulties of access to public spaces for disabled people, who are at best tolerated but can never be truly at ease as the potential for an encounter always looms. In considering how relational encounters and their associated emotions shape experiences of supposedly “accessible” spaces we need to rethink what we mean by access. As Titchkosky (2011) reminds us, getting people in is only half the issue. The affective impact of encounters means that even if they are no physical barriers, due to the impact of psycho-emotional barriers disabled people still cannot gain access to public spaces on the same terms as others. Rather, there is a significant cost of emotion work and energy needed to simply exist in public. It is no surprise then, that “going out in public so often takes courage. How many of us find that we can't dredge up the strength to do it day after day, week after week, year after year, a lifetime of rejections and revulsion?” (Morris, 1991, p. 25).

My participants' experiences also highlight the importance of considering the wider cultural and political context in which encounters take place. Many of my participants' encounters were explicitly shaped by the specific British context of over a decade of austerity politics, which at the time of writing is set to continue with further plans for disability welfare reform by the new Labour government (Helm, 2024). This results in prejudice and resentment against disabled people based on the false and harmful binary of the many “fakers” or “scroungers” vs. the few “deserving” ones (Briant et al., 2013). The resulting negative affects towards disability stick to disabled people, becoming stronger over time (Ahmed, 2001). While the Blue Badge accessible parking scheme is not directly linked to the welfare system and has been largely exempted from cuts and associated negative media coverage in the UK, this “stickiness” means my participants nevertheless experienced these negative attitudes in parking spaces. While “scrounger” rhetoric persists in politics and media and encourages the public to police disabled people's behaviour in parking spaces, most disabled people risk facing hostility when in public space.

As well as sticking to disabled bodies and spaces, the negative affects associated with accessible parking encounters can also shift between people, as highlighted by my participant who felt anxiety based on storeys she had heard from other people. While Reeve (2008) posits that psycho-emotional disablism occurs primarily in the private sphere whereas structural disablism happens in the public sphere, I argue that the pervasive “stickiness” of affects blurs the boundaries between the public and private. The wider abjection of disabled people in public discourse is replicated in encounters, meaning they are never just individual experiences, but rather reminders of the wider hostility and abjection in society. Anxiety about potential confrontation is a daily reality for many disabled people and these negative affects circulate in public spaces (Burch, 2021), meaning that the psycho-emotional disablism does not happen purely on an individual or personal level. Rather, the sharing of these experiences with others is, for better or worse, an integral aspect of encounters. While storeys from others can contribute to anxieties, sharing our everyday experiences with other disabled people can also be liberating and an expression of solidarity. As Keith (1996) highlights, swapping storeys about encounters is often the first thing disabled people do when we meet. Several of my interview participants also described being able to speak about their experiences (both during the interviews themselves and more generally with others in the disability community) as cathartic. Many were also connected with other disabled people through social media or through Disabled People's Organisations. As Summers-Effler (2002) argues, solidarity with others can be crucial in forming a collective and political identity as a disabled person, confirming to the disabled person that their experiences and the resulting emotions are reasonable, and understanding them as injustices done to them. For many of my participants, this solidarity was an essential survival mechanism for how they managed encounters and resisted the negativity found in accessible parking spaces.

The societal and communal affects attached to accessible parking encounters, then, frame the difficult and highly emotional decisions disabled people must make to navigate everyday public life. While my initial aim was to explore in detail the management strategies disabled people employed, it quickly became clear during the interviews that the difficult emotions my participants felt, as well as the work they put in to navigate them, were very similar despite the different strategies employed. While some participants were highly conflict-avoidant, others tended to be more assertive and even “belligerent” (a term my participant Frank used to describe himself). It thus becomes clear that there are no right or wrong ways to navigate encounter. Rather, in line with the social-relational model of disability (Thomas, 1999), disabled people in public are being “disabled” by other people's attitudes and assumptions. The social-relational model of disability's focus on the role of interpersonal interactions therefore facilitates a radical approach to disability by exposing how disability is not just about impersonal and static barriers such as steps. Rather, it is also something that is actively done *to* us by other people, in the same way as other marginalised groups experience prejudice and oppression. Indeed, for many of my participants with multiple marginalised identities, these experiences intersected with other forms of discrimination they experienced; for example, Amir highlighted how he could never be sure that his encounters were not also racially motivated. While marginalised groups often face being accused of overreacting and even being pathologized as paranoid when expressing their fear of discrimination and oppression (Schalk, 2018), exploring the context of encounters highlights that the anxiety and anger felt by my participants in these spaces are not unreasonable at all. Rather, these emotions are a perfectly logical reaction to the hostility and discrimination faced by disabled people on a daily basis (Morris, 1991; Reeve, 2006).

In conclusion, this paper has made a contribution to the sociology of emotions by uncovering “the affective structures and the emotional dynamics of social reality” (Bericat, 2015, p. 499) in the context of disabled people's experiences of everyday psycho-emotional disablism arising from encounters with strangers. This leads to a more nuanced understanding of the role of affect in contributing to experiences of exclusion and oppression for marginalised groups. I have laid bare the daily work that goes into navigating public space and the emotional energy that is required by disabled people to make difficult decisions and navigate precarious interactions in order to access the same spaces that others take for granted. I have explored the affects of public encounters through the lenses of impression management, emotion work, and microaggressions, highlighting how disabled people are required to manage both themselves and others and put considerable work into assessing the situation to ascertain the risk of an escalation. The title of this paper, “walking on eggshells” helps to visualise the careful balance disabled people have to strike between appeasing others and standing up for themselves. I have also examined how negative discourses around disability and welfare fraud lead to suspicion of disabled people in public, particularly for those who are incongruous with a stereotype of disability, and explored some of the intersectional concerns in these stereotypes. The resulting (self-)surveillance means disabled people can never be unwatched in public, and by having to perform both impression management and emotion work, disabled people need to spend considerable emotional energy to survive in public. While everyday encounters are often low-level incidents rather than outright hate crimes (Burch, 2021; Hall, 2019), they nevertheless have a cumulative emotional impact on the disabled person, reflecting the abjection and prejudice that persists against disabled people at a societal level. By drawing attention to encounters as a substantial barrier to disabled people's participation in public life, this paper has highlighted how disability is “constituted by and between people” (Titchkosky, 2005, p. 220). Through focusing on this impact and examining the psycho-emotional disablism (Reeve, 2008) that occurs as a result of encounters with strangers, we can thus better understand realities of everyday oppression faced by disabled people. While negative attitudes and emotions towards disability persist and stick to disabled bodies, there can be no truly equal access even in supposedly accessible spaces.

## Data Availability

The datasets for this article are not publicly available due to concerns regarding participant/patient anonymity. Requests to access the datasets should be directed to the corresponding author.

## Footnotes

^1^In solidarity with other queer disability scholars, I have made an explicit choice not to cite work published in the *Disability and Society* journal after 2018, given its executive editor's anti-trans stance (Slater and Liddiard, 2018).

## References

[B1] Åhäll L. (2018). Affect as methodology: feminism and the politics of Emotion. Int. Pol. Sociol. 12, 36–53. 10.1093/ips/olx024

[B2] Abrams T. (2014). Re-reading erving goffman as an emancipatory researcher. Disabil. Stud. Q.34. 10.18061/dsq.v34i1.3434

[B3] Ahmed S. (2000). Strange Encounters: Embodied Others in Post-Coloniality. London, New York: Routledge.

[B4] Ahmed S. (2001). The Organisation of Hate. Law Critique 12, 345–365. 10.1023/A:1013728103073

[B5] Ahmed S. (2014). The Cultural Politics of Emotion Edinburgh: Edinburgh University Press.

[B6] Aidley D. Fearon K. (2021). Doing Accessible Social Research: A Practical Guide. Bristol: Bristol Policy Press.

[B7] Bericat E. (2015). The sociology of emotions: four decades of progress. Curr. Sociol. 64, 491–513. 10.1177/0011392115588355

[B8] Berlant L. G. (2011). Cruel Optimism. Durham, NC: Duke University Press.

[B9] Braun V. Clarke V. (2006). Using thematic analysis in psychology. Qual. Res. Psychol. 3, 77–101. 10.1191/1478088706qp063oa

[B10] Braun V. Clarke V. (2021). One size fits all? What counts as quality practice in (reflexive) thematic analysis? Qual. Res. Psychol. 18, 328–352. 10.1080/14780887.2020.1769238

[B11] Braun V. Clarke V. (2022). Thematic Analysis: A Practical Guide. Los Angeles: Sage.

[B12] Briant E. Watson N. Philo G. (2013) Reporting disability in the age of austerity: the changing face of media representation of disability disabled people in the United Kingdom the creation of new “folk devils'. Disabil. Soc. 28, 874–889. 10.1080/09687599.2013.813837

[B13] Brown L. C. (2013). “Stigma: an enigma demystified,” in: *The Disability Studies Reader* (4th Edn.), ed. L. J. Davis (New York: Routledge), 147–160.

[B14] Burch L. (2021). Understanding Disability and Everyday Hate. Cham: Springer International Publishing AG.

[B15] Burr V. (2015). Social Constructionism. London, New York: Routledge.

[B16] Cahill S. E. Eggleston R. (1994). Managing emotions in public: the case of wheelchair users. Soc. Psychol. Q. 57, 300–312. 10.2307/2787157

[B17] Cahill S. E. Eggleston R. (1995). Reconsidering the stigma of physical disability: wheelchair use and public kindness. Sociol. Q. 36, 681–698. 10.1111/j.1533-8525.1995.tb00460.x

[B18] Coleman-Fountain E. McLaughlin J. (2013). The interactions of disability and impairment. Soc. Theor. Health 11, 133–150. 10.1057/sth.2012.21

[B19] Collins K. M. T. (2015). “Validity in multimethod and mixed research,” in The Oxford Handbook of Multimethod and Mixed Methods Research Inquiry, eds. S. N. Hesse-Biber and R. B. Johnson (Oxford, New York: Oxford University Press), 240–256.

[B20] Cornelius L. J. Harrington D. (2014). “Why social justice research? Giving voice to the unheard,” in A Social Justice Approach to Survey Design and Analysis, eds. L. J. Cornelius and D. Harrington. New York: Oxford University Press.

[B21] Cram F. Mertens D. M. (2015). “Transformative and indigenous frameworks for multimethod and mixed methods research,” in The Oxford Handbook of Multimethod and Mixed Methods Research Inquiry, eds. S. N. Hesse-Biber and R. B. Johnson (Oxford, New York: Oxford University Press), 91–119.

[B22] Creswell J. W. Plano Clark V. L. (2018). Designing and Conducting Mixed Methods Research. Los Angeles: Sage.

[B23] Deal M. (2003). Disabled people's attitudes toward other impairment groups: a hierarchy of impairments. Disabil. Soc. 18, 897–910. 10.1080/0968759032000127317

[B24] Egner J. (2017). “A messy trajectory: from medical sociology to crip theory,” in Sociology Looking at Disability: What Did We Know and When Did We Know It, eds. S. E. Green and S. N. Barnartt (Bingley: Emerald Group Publishing Limited), 159–192.

[B25] Finkelstein V. (1998). “Emancipating disability studies,” in The Disability Reader: Social Science Perspectives, ed. T. Shakespeare (London: Continuum), 28–49.

[B26] Flynn S. (2017). Engaging with materialism and material reality: critical disability studies and economic recession. Disabil. Soc. 32, 143–159. 10.1080/09687599.2017.1284650

[B27] Foucault M. (1991). Discipline and Punish: The Birth of the Prison. London: Penguin Books.

[B28] French S. (1994). “The disabled role,” in On Equal Terms: Working with Disabled People, ed. S. French (Oxford: Butterworth-Heinemann), 47–60.

[B29] Garland-Thomson R. (1997). Extraordinary Bodies: Figuring Physical Disability in American Culture and Literature. New York: Columbia University Press.

[B30] Garland-Thomson R. (2006). Ways of staring. J. Vis. Cult. 5, 173–192. 10.1177/1470412906066907

[B31] Garthwaite K. (2011). “The language of shirkers and scroungers?' Talking about illness, disability and coalition welfare reform. Disabil. Soc. 26, 369–372. 10.1080/09687599.2011.560420

[B32] Goffman E. (1972). Relations in Public: Microstudies of the Public Order. New York, London: Harper and Row.

[B33] Goffman E. (1986). Stigma: Notes on the Management of Spoiled Dentity. New York: Touchstone.

[B34] Goffman E. (1990). The Presentation of Self in Everyday Life. London: Penguin.

[B35] Gorton K. (2007). Theorizing emotion and affect: feminist engagements. Feminist Theor. 8, 333–348. 10.1177/1464700107082369

[B36] Halberstam J. (2011). The Queer Art of Failure. Durham, NC: Duke University Press.

[B37] Halberstam J. (2018). Female Masculinity. Durham; London: Duke University Press.

[B38] Hall E. (2019). A critical geography of disability hate crime. Area 51, 249–256. 10.1111/area.12455

[B39] Hall E. (2024). “Geographies of disability hate crime,” in Disability Hate Crime: Perspectives for Change, ed. L. Burch and D. Wilkin (Oxford: Taylor and Francis Group), 43–58.

[B40] Hall E. Bates E. (2019). Hatescape? A relational geography of disability hate crime, exclusion and belonging in the city. Geoforum 101, 100–110. 10.1016/j.geoforum.2019.02.024

[B41] Hall E. Wilton R. (2017). Towards a relational geography of disability. Prog. Hum. Geogr. 41, 727–744. 10.1177/0309132516659705

[B42] Hankivsky O. Grace D. (2015). “Understanding and emphasizing difference and intersectionality in multimethod and mixed methods research,” in The Oxford Handbook of Multimethod and Mixed Methods Research Inquiry, eds. S. N. Hesse-Biber and R. B. Johnson (Oxford, New York: Oxford University Press), 110–127.

[B43] Helm T. (2024). Pressure mounts on Rachel Reeves to drop “dangerous' £1.3bn cut to benefits for disabled. The Observer (October12, 2024).

[B44] Hesse-Biber S. N. (2015). “Introduction: navigating a turbulent research landscape: working the boundaries, tensions, diversity, and contradictions of multimethod and mixed methods inquiry,” in The Oxford Handbook of Multimethod and Mixed Methods Research Inquiry, eds. S. N. Hesse-Biber and R. B. Johnson (Oxford, New York: Oxford University Press), xxxiii–liii.

[B45] Hesse-Biber S. N. Griffin A. J. (2015). “Feminist approaches to multimethod and mixed methods research: theory and praxis,” in: *The Oxford Handbook of Multimethod and Mixed Methods Research Inquiry*, eds. S. N. Hesse-Biber and R. B. Johnson (Oxford, New York: Oxford University Press), 72–90.

[B46] Hochschild A. R. (1979). Emotion Work, Feeling Rules, and Social Structure. Am. J. Sociol. 85, 551–575. 10.1086/227049

[B47] Hochschild A. R. (2020). The Managed Heart: Commercialization of Human Feeling. Berkeley, CA: University of California Press.

[B48] Hollomotz A. (2013). “Disability and the continuum of violence,” in Disability, Hate Crime and Violence, eds. A. Roulstone and H. Mason-Bish (London, New York: Routledge), 52–63.

[B49] Hughes B. (2007). Being disabled: towards a critical social ontology for disability studies. Disabil. Soc. 22, 673–684. 10.1080/09687590701659527

[B50] Hughes B. (2015). Disabled people as counterfeit citizens: the politics of resentment past and present. Disabil. Soc. 30, 991–1004. 10.1080/09687599.2015.1066664

[B51] Imrie R. (2001). Barriered and bounded places and the spatialities of disability. Urban Stud. 38, 231–237. 10.1080/00420980124639

[B52] Kafer A. (2013). Feminist, Queer, Crip. Bloomington, Indiana: Indiana University Press.

[B53] Keith L. (1996). “Encounters with strangers: the public's responses to disabled women and how this affects our sense of self,” in Encounters with Strangers: Feminism and Disability, ed. J. Morris. London: Women's Press.

[B54] Keller R. M. Galgay C. E. (2010). “Microaggressive experiences of people with disabilities,” in Microaggressions and Marginality: Manifestations, Dynamic, and Impact, ed. D. W. Sue (Hoboken, N.J: Wiley), 241–66. 10.1037/e615712009-001

[B55] Lefebvre H. (1991). The Production of Space. Oxford: Blackwell.

[B56] Lipman C. (2006). The emotional self. Cult. Geogr. 13, 617–624. 10.1191/1474474006cgj378ra

[B57] Manji K. (2017). Social security reform and the surveillance state: exploring the operation of “hidden conditionality' in the reform of disability benefits since 2010. Soc. Pol. Soc. 16, 305–314. 10.1017/S147474641600052X

[B58] McEnhill L. Byrne V. (2014). 'Beat the cheat': portrayals of disability benefit claimants in print media. J. Pov. Soc. Just. 22, 99–110. 10.1332/175982714X13971346086512

[B59] Mclaughlin J. Coleman-Fountain E. (2018). “Pursuit of ordinariness: dynamics of conforming and resisting in disabled young people's embodied practices,” in Disability, Normalcy, and the Everyday, eds. G. M. Thomas and D. Sakellariou (London: Routledge), 61–83.

[B60] McRuer R. (2002). “Compulsory able-bodiedness and queer/disabled existence,” in Disability Studies: Enabling the Humanities, eds. S. L. Snyder, B. J. Brueggemann and R. Garland-Thomson (New York: Modern Language Association of America), 88–99.

[B61] Meekosha H. Shuttleworth R. (2009). What's so 'critical' about critical disability studies? Aust. J. Human Rights 15, 47–75. 10.1080/1323238X.2009.11910861

[B62] Morrigan C. (2017). Trauma time: the queer temporalities of the traumatized mind. Somatechnics 7, 50–58. 10.3366/soma.2017.0205

[B63] Morris J. (1991). Pride Against Prejudice: A Personal Politics of Disability. London: Women's Press.

[B64] Morris J. (1992). Personal and political: a feminist perspective on researching physical disability. Disabil. Handicap Soc. 7, 157–166. 10.1080/02674649266780181

[B65] Nittle N. (2019). Recognizing Microaggressions. New York: Enslow Publishing.

[B66] Oliver M. (1996). “A sociology of disability or a disablist sociology?,” in Disability and Society: Emerging Issues and Insights, ed. L. Barton (London: Longman), 18–42.

[B67] Piggott L. (2011). Prosecuting disability hate crime: a disabling solution? People Place Policy. 5, 25–34. 10.3351/ppp.0005.0001.0003

[B68] Plano Clark V. L. Ivankova N. V. (2016). Mixed Methods Research: A Guide to the Field. Los Angeles: SAGE.

[B69] Pritchard E. (2021). Dwarfism, Spatiality and Disabling Experiences. London; New York: Routledge.

[B70] Ralph S. Capewell C. Bonnett E. (2016) Disability hate crime: persecuted for difference. Br. J. Special Educ. 43, 215–232. 10.1111/1467-8578.12139

[B71] Reeve D. (2004). “Psycho-emotional dimensions of disability and the social model,” in Implementing the Social Model of Disability, ed. C. Barnes and G. Mercer (Leeds: Disability Press*)*, 83–100.

[B72] Reeve D. (2006). “Towards a psychology of disability: the emotional effects of living in a disabling society,” in Disability and Psychology: Critical Introductions and Reflections, ed. D. Goodley and R. Lawthom (London: Macmillan Education), 94–107. 10.1007/978-1-137-12098-4_7

[B73] Reeve D. (2008). Negotiating Disability in Everyday Life: The Experience of Psycho-Emotional Disablism. Lancaster University, Lancaster.

[B74] Reeve D. (2014). “Part of the problem or part of the solution? How far do “reasonable adjustments' guarantee “inclusive access for disabled customers?',” in: *Disability, Spaces and Places of Policy Exclusion*, eds. K. Soldatic, H. Morgan and A. Roulstone (Abingdon: Routledge), 99–114.

[B75] Reeve D. (2015). “Psycho-emotional disablism in the lives of people experiencing mental distress,” in Madness, Distress and the Politics of Disablement, eds. H. Spandler, J. Anderson and B. Sapey (Bristol: Policy Press), 99–112. 10.2307/j.ctt1t898sg.12

[B76] Schalk S. (2018). Bodyminds Reimagined: (Dis)ability, Race, and Gender in Black Women's Speculative Fiction. Durham, NC: Duke University Press.

[B77] Schalk S. Kim J. B. (2020). Integrating race, transforming feminist disability studies. Signs J. Women Cult. Soc. 46, 31–55. 10.1086/709213

[B78] Scully J. L. (2010). Hidden labor: Disabled/Nondisabled encounters, agency, and autonomy. Int. J. Fem. Approach. Bioeth. 3, 25–42. 10.3138/ijfab.3.2.25

[B79] Shakespeare T. (1996). “Power and prejudice: issues of gender, sexuality and disability,” in *Disability and Society: Emerging Issues and Insights*, ed. L. Barton (London: Longman), 191–224.

[B80] Shildrick M. Price J. (1996). Breaking the boundaries of the broken body. Body Soc. 2, 93–113. 10.1177/1357034X96002004006

[B81] Siebers T. (2008). Disability Theory. Ann Arbor, MI: University of Michigan Press.

[B82] Slater J. (2013). Constructions, Perceptions and Expectations of Being Disabled and Young: A Critical Disability Perspective. Manchester Metropolitan University, Manchester.

[B83] Slater J. Jones C. (2021). Toilet signs as border markers: exploring disabled people's access to space. Int. J. Disabil. Soc. Just. 1, 50–72. 10.13169/intljofdissocjus.1.1.0050

[B84] Slater J. Liddiard K. (2018). Why disability studies scholars must challenge transmisogyny and transphobia. Can. J. Disabil. Stud. 7, 83–93. 10.15353/cjds.v7i2.424

[B85] Sue D. W. (2010). Microaggressions in Everyday Life: Race, Gender, and Sexual Orientation. Hoboken, NJ: Wiley.

[B86] Summers-Effler E. (2002). The micro potential for social change: emotion, consciousness, and social movement formation. Sociol. Theor. 20, 41–60. 10.1111/1467-9558.00150

[B87] Thomas C. (1999). Female Forms: Experiencing and Understanding Disability. Buckingham: Open University Press.

[B88] Thomas G. M. Sakellariou D. (2018). “Introduction: disability normalcy and the everyday,” in Disability, Normalcy, and the Everyday, eds. G. M. Thomas and D. Sakellariou (London: Routledge*)*, 3–16.

[B89] Timm R. (2002). Disability-Specific Hassles: The Effects of Oppression on People with Disabilities. San Francisco Bay, Ann Arbor: Alliant International University.

[B90] Titchkosky T. (2005). “Looking blind: a revelation of culture's eye,” in Bodies in Commotion: Disability and Performance, eds. C. Sandahl and P. Auslander P (Ann Arbor: University of Michigan Press), 219–229.

[B91] Titchkosky T. (2011). The Question of Access: Disability, Space, Meaning. Toronto: University of Toronto Press Incorporated.

[B92] Trainor L. R. Bundon A. (2021). Developing the craft: reflexive accounts of doing reflexive thematic analysis. Qual. Res. Sport Exer. Health 13, 705–726. 10.1080/2159676X.2020.1840423

[B93] Tregaskis C. (2003). Constructions of Disability: Researching Inclusion in Community Leisure. Hoboken: Taylor and Francis.

[B94] Tyler I. (2013). Revolting Subjects: Social Abjection and Resistance in Neoliberal Britain. London; New York: Zed.

[B95] Tyler I. (2020). Stigma: The Machinery Of inequality. London: Zed.

[B96] Valentine G. (2008). Living with difference: reflections on geographies of encounter. Prog. Hum. Geogr. 32, 323–337. 10.1177/0309133308089372

[B97] Watermeyer B. Swartz L. (2008). Conceptualising the psycho-emotional aspects of disability and impairment: the distortion of personal and psychic boundaries. Disabil. Soc. 23, 599–610. 10.1080/09687590802328477

[B98] Wechuli Y. (2024). Passing as able-bodyminded, disabled, or supercrip: rethinking impression management strategies through a disability lens. Symbol. Interac. 10.1002/symb.711

[B99] Wetherell M. (2012). Affect and Emotion: A New Social Science Understanding. Los Angeles; London: Sage.

[B100] Wetherell M. (2015). Trends in the turn to affect: a social psychological critique. Body Soc. 21, 139–166. 10.1177/1357034X14539020

[B101] Wilkin D. (2020). Disability Hate Crime: Experiences of Everyday Hostility on Public Transport. London: Palgrave Macmillan.

[B102] Wilson H. F. (2017). On geography and encounter: Bodies, borders, and difference. Prog. Hum. Geogr. 41, 451–471. 10.1177/0309132516645958

